# Integrated care cannot be designed in Whitehall

**DOI:** 10.5334/ijic.814

**Published:** 2012-05-18

**Authors:** Ara Darzi, Peter Howitt

**Affiliations:** Paul Hamlyn Chair of Surgery, Imperial College, London, UK; Centre for Health Policy, Imperial College, London, UK

**Keywords:** England, GP-led health centres, integrated care, Integrated Care Pilots, policy

## Abstract

In recent years England has introduced a number of initiatives to promote more integrated care. Two contrasting examples are the GP-led health centres and the Integrated Care Pilots announced in the interim and final reports, respectively, of the NHS Next Stage Review in 2007–2008. The GP-led health centres were proposed as a very centralised, prescriptive approach where the aim was that all the NHS should adopt the same model of facilitating integration through co-location. Integrated Care Pilots, on the other hand, looked to the NHS to suggest their own solutions to improve integration, resulting in a variety of solutions tailored to the needs of localities. Although the results of the evaluation of the Integrated Care Pilots have been equivocal, this bottom-up approach must be the right way to foster integrated care. Long-term commitment to integrate care is needed, as well as more exploration of integration between primary care and hospitals.

## Introduction

In the last decade there has been a recognition amongst policy-makers in England that the most vulnerable can fall between the gaps in care provided by both different public services (e.g. health and social care) and by different parts of the health system [e.g. general practitioners (GPs) and hospitals]. Continuing attempts have been made to encourage integration to meet the needs of patients, especially those with long-term conditions such as diabetes. When I became a health minister in the last Labour Government my focus was on improving the quality of care in the NHS and one facet of this was how to encourage more integrated care. The best way to achieve this was far from clear and we consequently tried a number of approaches. In this article I want to contrast two different initiatives—GP-led health centres and Integrated Care Pilots—and the lessons they provide on how to foster integrated care.

## The wrong approach to integration—GP-led health centres

The announcement of 150 GP-led health centres was one of the centrepieces of the *Next Stage Review Interim Report* [1]. The GP-led health centres were, of course, not simply about integrated care—they were principally a response to public dissatisfaction with access to GPs, particularly outside the working day. It was envisaged, however, that the centres would contribute to integration by being hubs for a range of services including diagnostics, physiotherapy and social care as well as GP consultations. Instead of people having to make multiple trips to different locations, here would be a ‘one-stop shop’ for regular care needs. Whilst I still believe that GP-led health centres (which were labelled ‘Darzi centres,’ not always affectionately) with extended opening hours and a greater range of services are a good idea, the way they were introduced was not the right way to foster integrated care.

Although I made clear that “these centres will reflect local need and circumstance” [1, p. 25] the reality was that the initiative’s credibility was badly damaged by its top-down nature. The interim report was launched just as the government were considering calling a snap general election in October 2007, and the political pressure was to have something which benefitted everyone. Thus the decision to have 150 centres, one for each Primary Care Trust (the local units of NHS administration in England). This would mean that every part of the country could be said to be gaining new provision. Such a rigid imposition took no account of existing local developments, however; for instance London PCTs were required to have GP-led health centres, even though many were already implementing the more wide-ranging polyclinic model I had advocated in my review of health services in London [2]. Nor did it reflect actual need, as some PCTs could have benefitted from several such centres, whilst in others provision was adequate. Furthermore, the allocation of one health centre per-PCT may have appeared even-handed, but in reality PCT size varied massively from Hartlepool with 100,000 people to Hampshire with a population of 1.3 million.

It is a fallacy to assume that co-location equals integrated care—much more needs to be done to align cultures, processes and incentives. However, being in the same building can be a stimulus to integrated care; for instance when I visited the Barkantine Health and Well-Being Centre in Tower Hamlets (one of the first five polyclinics opened in London) I heard that greater co-operation between mental health staff and midwives on maternal mental health had been facilitated by them being co-located. The powerful spur of co-location was diluted, though, in the inflexible nature of the policy and the result was a missed opportunity to facilitate integration.

## The right approach to integration—Integrated Care Pilots

A better means to encourage integrated care was the concept of Integrated Care Organisations, outlined in *High Quality Care for All*, the final report of the NHS Next Stage Review [3]. Here the emphasis was on ‘empowering clinicians’ and ‘inviting proposals’ [3, p. 65] rather than requiring everywhere to adopt the same approach. By turning to the NHS (often in partnership with organisations such as local authorities and the police) to develop their own approaches to integrated care, the result was a range of creative solutions to local problems. In a competitive process 16 sites were chosen to become Integrated Care Pilots, the changed label recognising that a new organisational structure was not always required [4].

The Integrated Care Pilots varied considerably in the model of integrated care they were testing. Some took a disease specific approach, such as the pilot in Northumbria looking at Chronic Obstructive Pulmonary Disease. Others, like Torbay which focused on the over 65s, were looking at a population segment. Their scope was also highly varied with some moving well beyond traditional NHS functions (for instance in the Durham Dales pilot there was an emphasis on keeping warm through home improvements such as loft insulation, because this was identified as helping prevent illness) whilst others, such as Church View Medical Practice pilot and its virtual ward round involving primary and secondary care clinicians, sought to break down barriers within the NHS.

The consistent thread running through all the Integrated Care Pilots was that they were formed by a coalition of the willing, professionals wanting to improve care for the people they served. They therefore represented a ‘bottom-up’ approach to encouraging integrated care. Indeed, the recently published evaluation of the Integrated Care Pilots highlights that the staff involved in the pilots were positive about the benefits that the pilots had brought [5, p. 44].

Whilst this development was positive, the evaluation report did have surprising findings about reductions in the quality of patient experiences and increases in emergency admissions within the pilot sites. These results may be a function of time, since patients do not always respond well initially to changes in their care arrangements even if they are evidence-based, whilst two years is a short period to measure impact on emergency admissions. It would be interesting to see how these sites would have fared over a five or even ten-year time-scale. Unfortunately such long-term evaluations are virtually impossible in an NHS where priorities are constantly changing and reorganisations regularly occurring.

Improvements in care take time and I therefore hope the new Clinical Commissioning Groups in England that were introduced by the Health and Social Care Act 2012 [6] as the primary purchasers of healthcare services will continue the Integrated Care Pilot experiment. I also hope there will be more exploration of other types of integrated care. Most of the Integrated Care Pilots focused on horizontal integration (i.e. between health and social care in the community), rather than vertical integration (between primary and secondary care). This was perhaps because of the unintended consequences of national policy goals such as choice of hospital provider, introduced in the last decade in England, which militate against such integration. The Department of Health must consider how it can support all types of integrated care by removing barriers and giving the local NHS the freedom to innovate.

## Conclusions

Integrated care cannot be imposed from above. It is a product of good local working relationships. I hope that the new Clinical Commissioning Groups in England will build on the Integrated Care Pilot work and not abandon it as yesterday’s venture, because the benefits may be seen in the long-term. I would also like to see more vertical integration of primary and secondary care, as that should better align incentives and may be more effective in reducing emergency admissions. To facilitate this, government’s responsibility should be to create an environment conducive to locally developed integrated care, not to create universal models for integration unresponsive to local circumstances.

## About the authors

**Professor Darzi biography:** Professor Darzi is an eminent cancer surgeon. Following a review he led into the healthcare needs of London, he was ennobled and appointed to be a health minister in the Labour government in 2007. In his first year as a minister he carried out a review into the NHS called the Next Stage Review, culminating in a final report entitled *High Quality Care for All*. He then spent a further year in office implementing this review, which focused on improving the quality of care patients received, including initiatives to integrate care.

**Peter Howitt biography:** Peter Howitt is a policy-maker, on secondment to the Centre for Health Policy from the Department of Health. During his time in the department, Peter was involved in policy development for the *Our Health, Our Care, Our Say White Paper*, one of the first government documents to really recognise the need for more integrated care, and *High Quality Care for All.*
